# Properties of Cephalopod Skin Ommochromes to Inhibit Free Radicals, and the Maillard Reaction and Retino-Protective Mechanisms in Cellular Models Concerning Oxidative Stress, Angiogenesis, and Inflammation

**DOI:** 10.3390/antiox11081574

**Published:** 2022-08-15

**Authors:** Luján Lidianys María Lewis, Philipp Dörschmann, Charlotte Seeba, Tabea Thalenhorst, Johann Roider, Simon Bernard Iloki Assanga, Juan Carlos Gálvez Ruiz, Teresa Del Castillo Castro, Ema Carina Rosas-Burgos, Maribel Plascencia-Jatomea, Josafat Marina Ezquerra Brauer, Alexa Klettner

**Affiliations:** 1 Departamento de Investigación y Posgrado en Alimentos, Universidad de Sonora, Blvd. Luis Encinas y Rosales S/N, Col. Centro, Hermosillo 83000, Sonora, Mexico; 2 Department of Ophthalmology, University of Kiel, University Medical Center, Arnold-Heller-Str. 3, Haus 25, 24105 Kiel, Germany; 3 Department of Biological Chemical Sciences, Sonora University, Blvd. Luis Encinas y Rosales S/N, Col. Centro, Hermosillo 83000, Sonora, Mexico; 4 Department of Research on Polymers and Materials, Sonora University, Blvd. Luis Encinas y Rosales S/N, Col. Centro, Hermosillo 83000, Sonora, Mexico

**Keywords:** ommochromes, cephalopods, ferroptosis, glycative stress, retinal pigment epithelium, uveal melanoma, vascular endothelial growth factor, interleukin

## Abstract

Ommochromes are pigments of invertebrates that exhibit oxidative stress protection. The aim of this study was to investigate ommochromes extracted from cephalopod’s skin for their ability to inhibit age-related-macular degeneration (AMD)-related factors such as H_2_O_2_-induced and iron-dependent oxidative stress (ferroptosis and erastin), accumulation of advanced glycation end-products (AGEs), as well as vascular endothelial growth factor (VEGF), and inflammatory cytokines (interleukin 6 and interleukin 8) secretion. As cell systems, we used primary porcine retinal pigment epithelium (RPE), human retinal pigment epithelium cell line ARPE-19 and uveal melanoma cell line OMM-1. In vitro, ommochromes produced an antiglycation effect by the inhibition of fructosylation reaction. The ommochromes showed protective effects against erastin- induced cell death in ARPE-19. In addition, in long-term stimulation (7 days) ommochromes decreased constitutively secreted VEGF, as well as interleukin 6 and interleukin 8 induced by Poly I:C in primary RPE. No relevant effects were detected in OMM-1 cells. The effects are dependent on the cell system, time of exposition, and concentration. This substance is of interest for further research concerning age-related macular degeneration.

## 1. Introduction

Ommochrome pigments are mainly present in the eyes of invertebrates (ommatidia and rhabdoms), insects and crustaceans, the cuticle of arthropods, and the skin of cephalopods, among others. Cephalopods, in particular the octopus (*Octopus vulgaris*) and squid (*Berryteuthis magister*), are considered one of the most sophisticated systems in nature, they can alter their appearance or body pattern showing a wide range of camouflage due to their chromatophores that contain nanostructured granules of ommochrome pigments. Chromophores pigments are the main products of tryptophan’s metabolic oxidation pathway and among them are kynurenine, kynurenic acid, 3-hydroxykynurenine, xanthommatin, and ommatin D [1,2].

The structure of the ommochromes is classified on a phenoxazone ring (the ketone-derivative of phenoxazine; heteroatoms N and O, low molecular weight, thermo-labile, light colored: ommatins) or phenothiazine ring (heteroatoms N and S, high molecular weight, thermoresistant, and related to intense colorations: ommins/ommidins). Structural changes of ommatins are based on ring substitutions (OR): dihydroxan-tommatin (R = H reduced form), rhodommatin (R = β-glucosyl-), ommatin D (R = SO_3_H), and decarboxylated xanthommatin (COOH removed) [1,2,3]. The precise structure has been proposed only for a few ommochromes, mainly from mass spectrometry de-termination. The structural diversity of ommochromes may be high, which may be related to the extraction methods (e.g., phenoxazone ring opening upon light exposition or hydrolysis in acidic conditions). Complete structural characterization of ommochromes is challenging due to their structural diversity and complexity. In addition, their low solubility adds to the challenge of their characterization [4,5].

These aromatic compounds grouped into ommatins, ommins, and ommidins not only produce color change in cephalopods but are also involved in various metabolic and physiological processes and provide protection against ultraviolet (UV) light for photoreceptor cells [2,6,7,8,9]. Ommochromes have a stable electron spin resonance (ESR) signal with a high concentration of paramagnetic (antiradical) centers, which increase with UV and visible light. Their chemical properties make them suitable as antioxidants by reacting with free radicals and preventing the oxidation of biomolecules such as lipids [3,7,10,11,12,13].

The retina of the vertebrate eye is constantly subjected to oxidative stress because of the constant exposure to highly energetic visible light, high oxygen tension, and considerable production of hydrogen peroxide (H_2_O_2_) in the mitochondria of photoreceptors and cells of the retinal pigment epithelium (RPE) [14,15,16]. In addition, because of the abundance of membranes in the photoreceptor outer segment, the retina is prone to lipid peroxidation [17]. Lipid peroxidation has been recognized as an inductor of ferroptosis, so-called iron-dependent cell death, characterized by an accumulation of lipid hydroperoxides [18,19]. Furthermore, in RPE cells, N-retinyl-N-retinylidene ethanolamine (A2E) is one of the major fluorescent pigments contained in lipofuscin granules or the so-called “age pigment” associated to elevated synthesis of proinflammatory cytokines and reactive oxygen species (ROS) [17]. The glycation reaction produces A2E using two molecules of different all-trans-retinal and amino group of a lipid of photoreceptor outer segment membrane, phosphatidylethanolamine [20].

Oxidative stress is a major contributor to the pathogenesis of age-related macular de-generation (AMD), including glycative stress [21,22,23]. AMD is the main cause of severe visual impairment in the elderly in the industrialized world with its incidence on the rise because of the demographic shift and changes in life style [24,25]. Clinically, AMD presents in an asymptomatically early form and two distinct late forms, the atrophic (or dry) form, and the exudative (or wet) AMD, in which pathologic vessels growth from beneath or into the retina [26]. On a cellular level, the pathology of AMD takes place between the light-sensitive photoreceptors, the underlying RPE which maintains the photoreceptors, and the choroid which supplies oxygen and nutrients to these cells [27]. AMD is a multifactorial disease with old age, genetic disposition, and environmental factors considered as major risk factors [28]. Pathological pathways include oxidative stress, inflammation and, in the case of the exudative form of AMD, pro-angiogenic signaling [21,29,30]. Oxidative stress, as pointed out above, is a constant condition in the retina. The RPE protects the retina from oxidative stress-induced damage [31]; however, this ability decreases during aging, leading to a reduced activation of oxidative stress pathways, RPE degeneration, and AMD-like features in animal models [32,33,34]. In particular, lipid peroxidation has been implicated in AMD pathogenesis, inducing ferroptotic cell death and chronic inflammation [35,36].

Chronic inflammation that persists due to constant degenerative and pro-inflammatory stimuli is considered a major factor in AMD development [22,30]. The RPE expresses toll-like receptors (TLR) to detect danger-associated molecular patterns and can react to these signals by secreting pro-inflammatory cytokines, which has also been implied to be contributing to the AMD development [37,38]. Among the cytokines that are increased by pro-inflammatory signaling are interleukin 6 (IL-6) and interleukin 8 (IL-8) [37,39]. In addition to its pro-inflammatory functions, IL-8 is also considered as pro-angiogenic cytokine [40].

While oxidative stress and inflammation are considered stress factors for all forms of AMD, angiogenesis is the major additional pathogenic mechanisms in exudative AMD. In exudative AMD, pathological vessels grow from the choroid beneath and into the retina [41]. These vessels are generally immature and leaky, causing edema, hemorrhages [37], and, in late-stage disease, a fibrotic scar [41,42]. These changes lead to a rapid deterioration of the tissue, causing this subtype of AMD to be responsible for the majority of severe vision loss in AMD [43]. While angiogenesis is a complex process involving a plethora of factors, the most important factor for angiogenesis both in health and disease is vascular endothelial growth factor (VEGF) [44,45]. VEGF is involved in angiogenesis in several cancers as well as in a variety of retinal diseases [29,45]. There are several sources of VEGF in the retina, but with regards to AMD, the RPE is considered the most important [27]. VEGF is secreted constitutively in order to protect the cells and to uphold the fenestration of the endothelium in the choroid, but its expression can be enhanced by a variety of factors, such as hypoxia, oxidative stress, or pro-inflammatory stimuli [46,47,48,49]. Indeed, the development of VEGF antagonists has profoundly changed the treatment modality in exudative AMD, quickly becoming the gold standard [50]. Unfortunately, while some visual gain can be achieved under anti-VEGF treatment, long-term treatment is needed which is usually accompanied by visual decline in the long run [51].

As mentioned above, the only available therapy for AMD targets VEGF and is applied after tissue degeneration and loss of visual acuity has already occurred. New therapies, targeting multiple pathways and those being able to be applied at an earlier stage of the disease are clearly warranted. Marine compounds may exhibit bioactivities which can attack multiple pathways involved in AMD development [52,53]. In this study, we investigate the effect of ommochromes derived from cephalopods on oxidative stress, ferroptotic cell death, inflammation, and VEGF secretion in ocular cells, focusing on the retinal pigment epithelium. Our data indicate that ommochromes may target several pathways of AMD development.

## 2. Materials and Methods

### 2.1. Materials

Octopus, *Octopus vulgaris* (*Octopodidae*, Incirrata) specimens, were caught off the coast of Bahía de Kino (Sonora, Mexico; 28°49′00″ N 111°56′00″ W, 15–18 °C) in November 2018 and January 2019. Squids *Berryteuthis magister* (*Gonatidae,* Oegopsina) were obtained frozen from a local market. All the samples were transported in plastic boxes with ice to the Marine Laboratory of the Food Research and Postgraduate Department (DIPA) of the University of Sonora and frozen at −20 °C for later use.

### 2.2. Isolation of Chromatophoric Granules and Pigment Extraction

The octopus’s samples were thawed at room temperature and the epidermal and dermal tissue contained in the chromatophore sacs were manually removed with the help of scissors and then were lyophilized at −46 °C (Labconco, Kansas City, MO, USA) for five days. The lyophilized skin was pulverized in a grinder (Krups, Solingen, Germany) and stored at 4 °C protected from light until the extraction process. The isolation of the chromatophoric granules and the extraction of the pigments (ommochromes) were carried out by means processes of solubilization of the octopus’s ommochromes in acidified methanol (1% *v*/*v* HCl) followed by successive steps of centrifugation and sonication.

To do this, 1 mg of lyophilized skin was added with 20 mL of the methanol–HCl. The mixture was kept under constant stirring for 10 min (Vortex, Barneveld, WI, USA) and sonicated in an ultrasonic bath (Branson BNS-M-1800, Branson Ultrasonics, Brookfield, CT, USA) for 10 min at room temperature 25 °C. The solution was centrifuged at 2000 rpm at 4 °C for 30 min (Sigma, Saint Louis, MO, USA) and the pigmented supernatant was collected. The extraction was repeated 3 times until no more color was collected. Finally, the pigmented supernatants were concentrated on a rotary evaporator (R-210, Buchi, Switzerland) at 37 °C [8].

Ommochromes were also isolated from the skin of fresh frozen Commander squid *Berryteuthis magister*. One hundred grams of finely cut squid skin was incubated at 5 °C in 200 mL of methanol for 24 h. The resulting solution was filtered on a paper filter, the filtrate was discarded, and 150 mL of methanol–HCl (0.5%) was added to the remaining material, stirred, and left for 24 h at 5 °C. The resulting solution of cherry-colored ommochromes was separated from the squid skin by filtration on a paper filter and neutralized 20% ammonia solution to pH 7.2–7.4. The ommochrome sediment was separated by centrifugation at 5000× *g* for 15 min. The resulting precipitate was redissolved in methanol–HCl (0.5%), and the ammonia precipitation procedure was repeated. Finally, the ommochrome precipitate was washed with distilled water and dried in a desiccator.

### 2.3. Structural Characterization of Ommochrome Extracts

#### 2.3.1. UV-Visible Spectrum and Fourier Transformed Infrared (FTIR) Spectroscopy

The ommochromes of octopus obtained with each type of solvent, methanol–HCl and methanol–acetic acid, were characterized by ultraviolet/visible absorption spectra (UV/vis) using a spectrophotometer (Varian Cary, 60 double beam) in a range of 200 to 600 nm with a range of 10.0 nm. The blank solution was 1% MeOH/HCl and 1% MeOH-CH_3_COOH, respectively, for each of the ommochrome extracts. In addition, Fourier transformed infrared (FTIR) spectroscopy was performed using a Perkin Elmer FTIR GX spectrometer (Waltham, MA, USA) applying 16 scans in the 4000–400 cm^−1^ spectral range. Spectra were measured using samples in KBr pellets.

#### 2.3.2. Nuclear Magnetic Resonance Spectroscopy (^1^H-RMN)

^1^H-NMR spectra was recorded on a Bruker Avance III 400 spectrometer (400 MHz), using DMSO-D6 as solvent. Chemical shifts are given in ppm relative to tetramethysilane (TMS).

#### 2.3.3. HPLC Analysis

Isolated ommochromes from squid were analyzed by high performance liquid chromatography (HPLC) using a Knauer chromatograph (Berlin, Germany) on a Diasphere 120 C18 column (4 × 250 mm; particle size, 5 μm). Solvent A was 10% aqueous acetonitrile containing 0.5% formic acid; solvent B was 100% acetonitrile containing 0.5% formic acid. The pigments were fractionated in a linear gradient (0–40%) of solution B in solution A for 60 min at a flow rate of 0.4 mL/min at 24 °C. Eluted pigments were registered with a Knauer K-2501 UV/Vis detector and an RF-10A-xl fluorescence detector (Shimadzu, Japan).

The ommochromes or standard compounds were dissolved in 100 μL of methanol containing 0.5% HCl. Tryptophan, kynurenine, 3-hydroxykynurenine, and xanthurenic acid (Sigma-Aldrich, Saint Louis, MO, USA) were used as standards. Xanthommatin was synthesized by autooxidation of 3-hydroxykynurenine. Absorption spectra were recorded with a Shimadzu UV–1601PC spectrophotometer. Fluorescence spectra were recorded with a Shimadzu RF-5301PC fluorimeter. The obtained data were processed with the RFPC version 2.0 software (Shimadzu, Kyoto, Japan).

### 2.4. Determination of Antioxidant Activity of Squid and Octopus Skin Ommochromes

#### 2.4.1. 2,2-Diphenyl-1-picrylhydrazyl (DPPH) Scavenging Activity

The capacity to scavenge the stable 2,2-Diphenyl-1-picrylhydrazyl (DPPH) radical (0.1 mM) of the ommochrome extracts from octopus was evaluated by the method of Brand-Williams et al. [54]. A range of concentration from 500 to 5000 µg/mL of the pigment extracts was used to quench the DPPH free radical and the mean inhibitory concentration (IC_50_) was calculated from percent scavenging (% antioxidant activity) through the equation:

% Antiradical Activity = (Ac − As)/Ac × 100%

As and Ac were the sample and control absorbances, respectively, measured in microplate spectrophotometer reader at 517 nm (Thermo Scientific Multiskan Spectrum, Waltham, MA, USA). Ascorbic acid was used as an antioxidant standard in a gradient concentration of 5–20 µg/mL.

#### 2.4.2. Ferric Reducing Antioxidant Power (FRAP)

The total antioxidant capacity of the octopus’s pigment extracts was evaluated using the FRAP assay [55]. The colorless oxidized Fe^3+^ form of iron converts to a blue-colored Fe^2+^ tri-pyridyl triazine (TPTZ)-reduced form, which is due to the action of the electron donation on antioxidants at a low pH. The colorimetric method was measured at 595 nm after 10 min the reaction in microplate spectrophotometer reader (Thermo Scientific Multiskan Spectrum). The working FRAP reagent was prepared immediately before to use by mixing 300 mM acetate buffer, pH 3.6, 10 mM TPTZ (2,4,6-tri(2-pyridyl)-*s*-triazine) in 40 mM HCl and 20 mM ferric chloride in proportions of 10:1:1 (*v*/*v*), respectively, 5 μL of samples diluted with 15 μL of PBS were added to 150 μL of FRAP reagent. The standard curve (100 a 2000 μM) was prepared with ascorbic acid and the results were expressed as μM equivalent of ascorbic acid.

#### 2.4.3. Quenching the Chemiluminescence of Luminol

Antioxidant activity was determined by quenching the chemiluminescence of luminol [56]. The value of the latent period of the ignition of chemiluminescence in the control and in the presence of various amounts of the squid ommochromes was used as a measured parameter. The kinetics of chemiluminescence was recorded on a Shimadzu RF 5301PC spectrofluorometer (Japan) at a luminescence wavelength of 470 nm and room temperature. The results were compared with the action of Trolox (6-hydroxy-2,5,7,8-tetramethylchroman-2-carboxylic acid), a water-soluble analogue of vitamin E, by measuring the dependence of the latent period of chemiluminescence development on the concentration of Trolox under the same conditions.

The reaction medium contained 0.05 M K-phosphate buffer, pH 7.4, 2.0 μM hemoglobin, 100 μM luminol, 100 μM EDTA, and various concentrations of squid ommochromes or Trolox as chemiluminescence quenchers. The reaction was started by adding 100 μM hydrogen peroxide. Either K-phosphate buffer or methanol HCl was used as a control. The control samples contained only the solvent (methanol–HCl) without ommochromes. The antioxidant activity of the ommochromes was expressed as the molar concentration of Trolox giving the same inhibitory effect as the ommochrome preparation containing 1 mg/mL dry matter.

### 2.5. Determination of Antiglycation Activity of the Ommochromes

Bovine serum albumin (BSA) was used as a glycation substrate, and fructose was used as a reducing sugar. It is known that fructose is 8–10 times more active than glucose in the formation of products of the Maillard reaction [57,58]. The incubation medium contained sterile 0.1 M potassium phosphate buffer, pH 7.4; 50 mM fructose, 4 mg/mL BSA, 2 mM sodium azide, and 40 μg/mL squid ommochromes in methanol–HCl. As control samples, we used samples that did not contain ommochromes, as well as samples containing ommochromes, but did not contain fructose. The samples were incubated at 37 °C in the dark with constant stirring for 3 days. After incubation, aliquots of samples were dialyzed against phosphate buffer to remove unreacted low molecular weight molecules. For dialysis, a Float-A-Lyser cellulose ether membrane (SPECTRUM Labs, Santa Clara, CA, USA) was used, which allows molecules with a molecular weight of less than 3.5 kDa to pass through. Dialysis was carried out for 20 h at 5 °C. After dialysis, the maximum fluorescence intensity of the modified albumin was measured at 435 nm (excitation wavelength 365 nm).

### 2.6. Cell Culture

The human uveal melanoma cell line OMM-1 [59] was kindly provided by Sarah Coupland and cultured in RPMI medium (PAN-Biotech, Aidenbach, Germany) with 10% fetal bovine Gibco^®^ serum (Thermo Fisher Scientific, Waltham, MA, USA) and 1% penicillin/streptomycin (PAN-Biotech).

Human immortal RPE cell line ARPE-19 [60] was bought from the American Type Culture Collection (ATCC) and cultured in DMEM medium (PAN-Biotech), supplemented with 1% penicillin/streptomycin (PAN-Biotech), 1% non-essential amino acids (PAN-Biotech), 2.5% HEPES (PAN-Biotech), and 10% fetal bovine Gibco^®^ serum (Thermo Fisher Scientific).

For the assays with cell lines, 200,000 cells/mL were seeded in 96-well plates and treated after one day of incubation at a confluence of 75–90%.

Primary porcine RPE cells were isolated from fresh pig eyes as previously described [60]. In brief, unnecessary tissue was cut off from the eye. The cornea, iris, ciliary body, and vitreous body was removed and the retina isolated. Dispatching of the RPE was conducted by trypsin and ethylenediaminetetraacetic acid incubation. Dispatched cells were put in DMEM (Pan-Biotech) with 2.5% HEPES (Pan-Biotech), 1% non-essential amino acids (Pan-Biotech), 1% penicillin/streptomycin (Pan-Biotech), and 10% fetal bovine Gibco^®^ serum (Thermo Fisher Scientific). Twelve eyes were collected and all the cells were seeded in 24-well plates and cultivated for two weeks until reaching confluence.

### 2.7. Cell Viability and Oxidative Stress Induction

OMM-1, ARPE-19, and RPE were treated with 1–100 µg/mL octopus’s ommochrome methanol–HCl extract, and cell viability was investigated after different time points (4 h and 24 h) to check for possible toxic effects. In addition, OMM-1 and ARPE-19 cells were treated with concentrations of 500 µM (OMM-1) or 250 µM (ARPE-19) H_2_O_2_ as well as 30 µM (OMM-1) or 20 µM (ARPE-19) erastin for 4 h and 24 h to induce oxidative stress.

Cell viability was determined with methyl thiazolyl tetrazolium (MTT) assay [61]. Therefore, cells were washed and treated with 0.5 mg/mL MTT agent (Sigma-Aldrich, St. Louis, MO, USA) for 2 h. After removing MTT, cells were dissolved in dimethylsulfoxide and absorbance was measured at 550 nm with spectrophotometer Elx800 (BioTek, Bad Friedrichshall, Germany).

### 2.8. VEGF Secretion

ARPE-19 and RPE cells were treated with 1–100 µg/mL of octopus’s ommochromes for the stated time periods (ARPE-19: three days; RPE: three and seven days), supernatants were collected for 24 h for ARPE-19 and 4 h for RPE. These supernatants were examined for secreted VEGF by VEGF DuoSet ELISA from R&D Systems (Minneapolis, MN, USA) as described in the instructor’s manual. Secreted VEGF in pg/mL was set in relation to the measured cell viability in % control.

### 2.9. Interleukin 6 and Interleukin 8 Secretion

Primary porcine RPE cells were treated with 50 µg/mL octopus’s ommochrome extract as well as 1 µg/mL LPS (Sigma-Aldrich), 10 µg/mL Poly I:C (Tocris BioScience, Bristol, UK) or 50 ng/mL TNFα (Tocris BioScience) for one, three and seven days [37]. Supernatants were collected for 24 h and interleukin 6 and interleukin 8 secretion was investigated with porcine IL-6 and IL-8 DuoSet ELISA Kit as described in the user manual.

### 2.10. Statistical Analysis

Normality was evaluated by the Shapiro–Wilk test. Because data were normally distributed, ANOVA test with consecutive student *t*-test for absolute data or a one-sample *t*-test for relative values was performed. *p* < 0.05 was considered statistically significant. All statistical analyses were performed with Microsoft Excel (Excel 2010, Microsoft, Redmond, WA, USA) and GraphPad Prism 9 (GraphPad Software, Inc., San Diego, CA, USA, 2021).

## 3. Results

### 3.1. Structural Characterization

**Figure 1:**
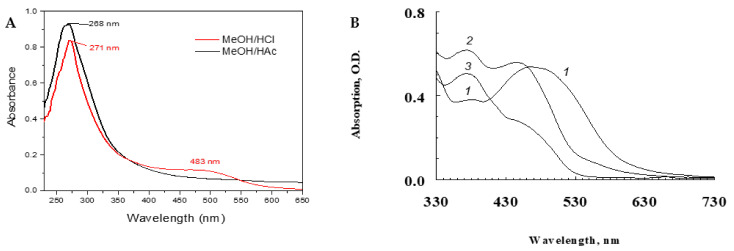
UV-Vis spectra of pigment extracts from the skin of *Octopus vulgaris* in methanol acidified HCl or acetic acid (**A**). Effect of hydrogen peroxide on the absorption spectrum of squid ommochromes in methanol–HCl (0.5%). 1—the original ommochromes spectrum, 2 and 3—oxidized ommochromes after adding 50 μL of 30% hydrogen peroxide to 2, 2 mL of the sample within 30 min of reaction (2), and after 20 h of reaction with hydrogen peroxide (3) (**B**).

To determine the presence of the ommochromes in the extracts of the pigments isolated from the chromatophore granules of the octopus, both extracts (methanol–HCl and methanol–acetic acid) were characterized by their absorbance spectra using UV-visible spectrophotometry (Figure 1). The ommochromes are commonly characterized by their absorbance spectra with three characteristic peaks at 256, 367, and 463 nm [2,3,6]. When evaluating the effect of pH on the redox potential of the acidified methanol, it was obtained that during extraction with MeOH/HCl (pH ≈ 1), mainly reduced ommochromes were isolated, and responsible for the red color unlike the oxidized ommochromes obtained in the MeOH/acetic acid extraction (pH ≈ 6), responsible for the yellow color. On the other hand, typical peaks of different ommochromes can occur in the visible region in the 430–520 nm range. In this work, a maximum of blue shift absorbance at λ_max_ 483 nm was observed in the supernatant of the red pigment extracted with MeOH/HCl, while this λ_max_ disappeared in MeOH/acetic acid with a yellow coloration (Figure 1A).

At the same time, the effect of hydrogen peroxide and exposure time on the oxidation of squid ommochromes was evaluated. In the UV-visible absorption spectrum, in the visible region, a maximum absorbance of reduced ommochromes was achieved near the 500 nm (spectrum (1)), but due to oxidation with H_2_O_2_ change, the absorption peaked at 430 nm (oxidized ommochromes, spectrum (2)). Similar behavior was observed to the effect of pH on the redox potential during the extraction with acidified methanol. When the ommochrome pigments were in reaction with H_2_O_2_ by 20 h (spectrum (3)) the maximum absorbance of λ_max_ at 430 nm was shifted to minimal adsorption (Figure 1B). This result indicates the possible degradation of ommochromes by hydrogen peroxide under long periods of time.

**Figure 2:**
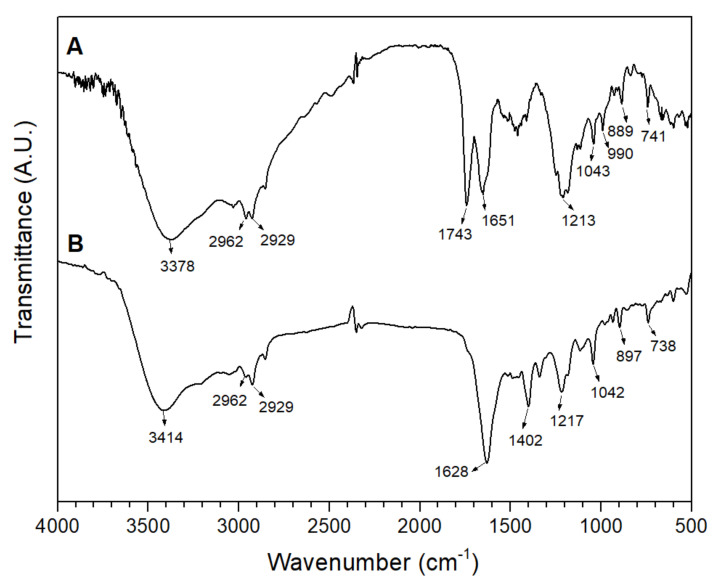
Infrared spectrum (FTIR) of pigments extracted in methanol–acids: (**A**) MeOH/HCl (1% *v*/*v*) and (**B**) MeOH/acetic acid (1% *v*/*v*).

The chemical structure of the octopus’s skin chromophores, associated with the methanol–acid extraction processes, was determined using FTIR spectroscopy. These spectra offer structural characterization by recognizing functional groups useful for identifying organic compounds from maximum absorption bands associated with ommochromic compounds [2,6]. An increase in the intensity of characteristic chemical groups of the ommochromes was observed, influenced by the type of solvent used in the extraction, which includes extension from 3018 to 3281 cm^−1^, 1575–1752 cm^−1^, 1152–1344 cm^−1^, and 1100–1300 cm^−1^ (Figure 2).

The broad profile of the bands suggested the contribution of various vibrational modes where aromatic rings and the characteristic N-H bond of the unique primary amine in xanthommatin contribute at 3378 and 3414 cm^−1^. The doublet at 1644 and 1709 cm^−1^ indicates the N-H scissor mode, associated with a weak vibration mode of the aromatic ketone groups (C=O) that extended towards 1768 cm^−1^. The typical of C=N of secondary amine of the pyridol ring at 1152−1344 cm^− 1^. Finally, the band of characteristic vibrational modes of C−N and C−O aromatic stretching, as well as C−C aromatic stretching in xanthommatin [3,6]. The carbomethoxy C=O (1768 cm^−1^) and quinonic C–O (1152–1344 cm^−1^) indicates that squid pigments contained ommochromes compounds of the xanthommatin-type.

**Figure 3:**
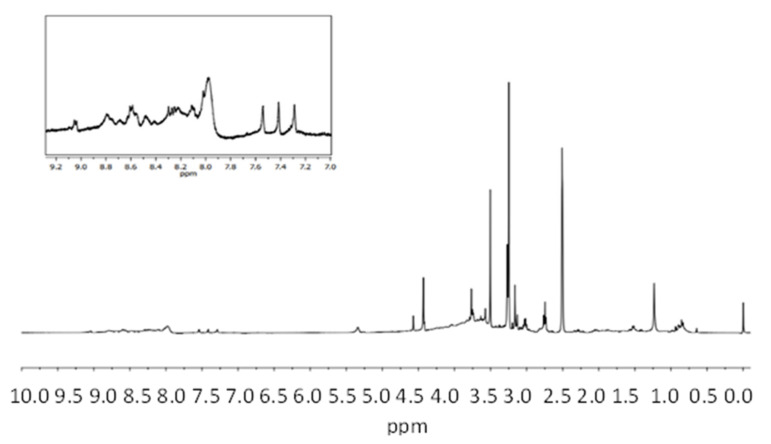
^1^H-NMR spectrophotometric spectra of octopus skin pigments extract with MeOH/HCl (1% *v*/*v*).

Data for the ^1^H-NMR spectrum of octopus skin pigment are reported as a chemical shift (δ ppm) (Figure 3) and compared with other works of the pigmented extracts from the squid and octopus skin and spectral databases. The signals corresponding to the functional groups in the phenoxazone ring are detected from δ 8.0–8.5 ppm that are assigned to aromatic protons at C8 and C9 basic structure of ommochrome. The absorption band at δ 4.5 ppm (triplet) is due to aromatic N-H protons that are representative of the amino groups of phenoxazone heterocycles. In addition, the two carbomethoxy groups appear as two singles at δ 3.25 and δ 3.5 ppm.

**Figure 4:**
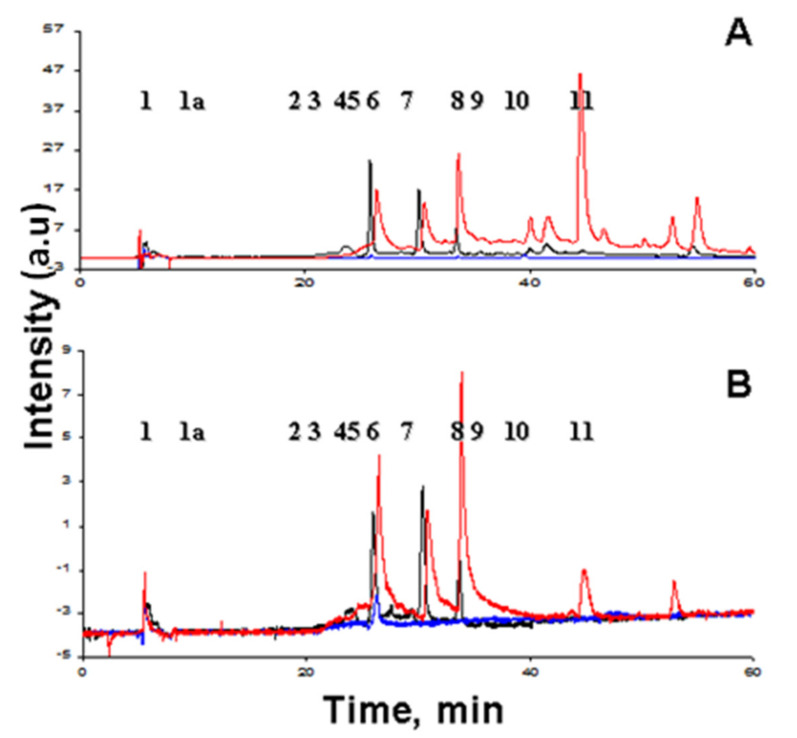
Comparative chromatography of initial (black), oxidized (blue) and reduced (red) ommochromes from squid skin; arbitrary units = a.u. (**A**) Detection by absorption at a wavelength of 490 nm. (**B**) Detection by fluorescence at a wavelength of 520 nm with excitation at a wavelength of 460 nm. Ommochromes were oxidized with hydrogen peroxide and reduced with ascorbic acid. Peaks: 1—xanthurenic acid, 2—decarboxylated xanthommatin, 4—xanthommatin.

From the data obtained by HPLC (Figure 4), xanthurenic acid, decarboxylated xanthommatin, and xanthommatin were found under detection by absorption (A) and by fluorescence (B). It can be seen that under the action of hydrogen peroxide, the squid’s ommochromes of the initial sample (black chromatogram) are oxidized (blue) and the peaks disappear in the interval of 20–35 min, and after the subsequent reduction (red) with ascorbate again appear on the chromatogram.

### 3.2. Antioxidant Activity of Squid and Octopus Skin Ommochromes

**Figure 5:**
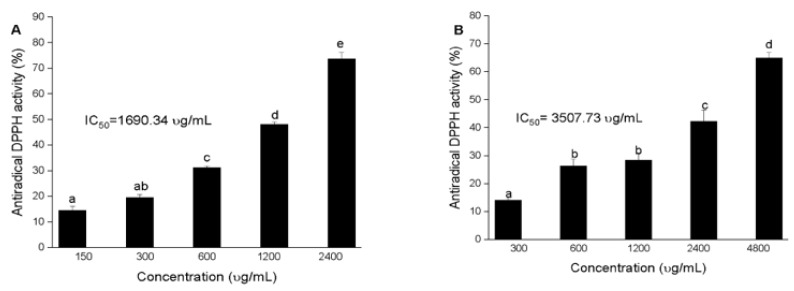
DPPH• antiradical activity by ommochromes from octopus of (**A**) MeOH/HCl (1% *v*/*v*) y (**B**) MeOH/acetic acid (1% *v*/*v*). Values are expressed as media ± S.D (*n* = 3). Different letters above the bar indicates the significant differences (*p* < 0.05), IC_50_ values mean inhibitory concentration (μg/mL) with significant differences (*p* < 0.05) by Tukey’s test for each extraction method.

The data obtained in this study revealed that the antioxidant activity of the pigments from octopus is concentration dependent. The highest antiradical DPPH activity was obtained in the extract of reduced ommochromes obtained in MeOH/HCl (IC_50_ = 1609.34 μg/mL) compared to the ommochromes extracted in MeOH/acetic acid (IC_50_ = 3507.73 μg/mL, *p* ≤ 0.05) (Figure 5). Since the extracting capacity of the antioxidant compounds depends on the polarity and redox potential of the extraction medium, a strong dependence of the antioxidant activity was observed on the type of solvent used. As can be seen in Figure 5, the average inhibition effect (IC_50_) of the DPPH radical varied between 1500 and 3500 μg/mL. However, the antioxidant capacity of these compounds was lower than that of ascorbic acid (11.10 μg/mL) used as a control. A smaller value indicates a greater antioxidant capacity because a smaller sample is required to achieve 50% discoloration of the DPPH control.

The ferric ion reducing antioxidant power (FRAP) of the two extracts exhibited a concentration-dependent activity. The values were in a range between 5 to 20 µM EAA/mg extract, significantly lower than the ascorbic acid control (5882.35 μmol/mg). The contribution of the redox potential of the solvents to the reducing power of the ommochromes showed that the compounds in methanol/HCl had the highest (*p* < 0.05) values of antioxidant activity, 14.76 μmol EAA/mg, with respect to the methanol/acetic acid (5.84 μmol EAA/mg). We can emphasize that the type of solvent used contributes in particular to the reducing power in the different extracts. These results are related to those obtained for the antiradical activity by DPPH.

**Figure 6:**
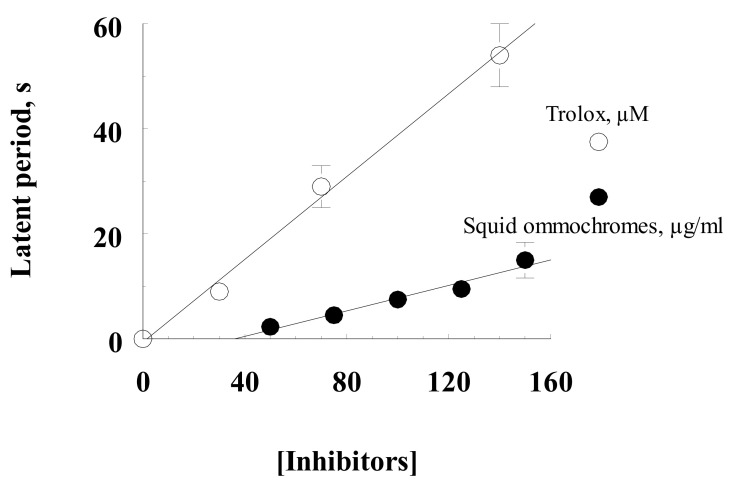
Comparative determination of antioxidant activity of squid skin ommochromes and Trolox by chemiluminescence method (by latency period). Ommochromes were dissolved in a mixture of methanol–HCl—K-phosphate buffer (1:1, *v*:*v*); Trolox was dissolved in 0.1 M K-phosphate buffer. Values are expressed as media ± S.D (*n* = 3), (*n*: number of independent experiments).

The main result of determining the antioxidant activity by quenching the chemiluminescence of luminol of squid skin ommochromes showed the dependence of the increase in the latent period of delay in the development of chemiluminescence on the concentration of inhibitors (Figure 6). Ommochromes of squid and Trolox were used as quenchers for chemiluminescence. Comparison of the antioxidant activity value with the value for the water-soluble vitamin E analogue Trolox showed that the squid skin ommochromes at a concentration of 1 mg/mL (1 g/L) exhibited the same inhibitory effect as 0.26 mM Trolox.

### 3.3. Determination of Antiglycation Activity of the Ommochromes

**Figure 7:**
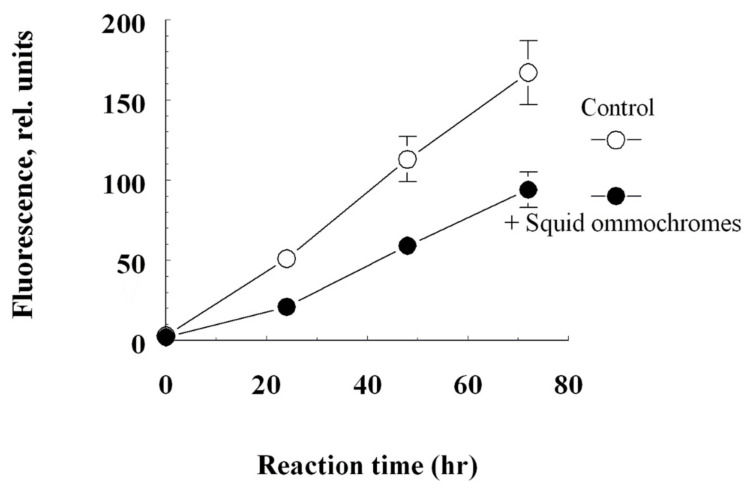
The inhibitory effect of the squid ommochromes on the fructosylation process of bovine serum albumin. Values are expressed as media ± S.D (*n* = 3), (*n*: number of independent experiments).

Ommochromes of squid skin exhibited an inhibitory effect on the BSA fructosylation reaction. It is well known that non-enzymatic glycation (Maillard reaction) under hyperglycemic conditions leads to the formation of so-called advanced glycation end products (AGEs) [20,62,63]. The ability of squid skin ommochromes to suppress the process of nonenzymatic glycation may be associated with their antioxidant activity. Figure 7 shows the kinetics of the increase in the fluorescent products of BSA modification in the control and in the presence of the squid ommochromes. As can be seen, the squid ommochromes at a concentration of 40 μg/mL led to a slowdown in the accumulation of fluorescent products in albumin.

### 3.4. Oxidative Stress and Ferroptosis Protection

**Figure 8:**
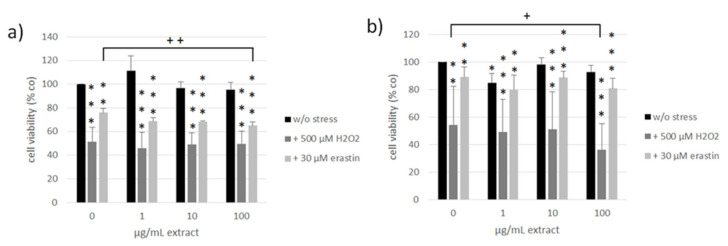
Cell viability of human uveal melanoma cell line OMM-1 treated with concentrations of ommochromes (1, 10, 100 μg/mL) and/or 500 µM H_2_O_2_ and 30 µM erastin for four (**a**) and 24 h (**b**) in % of the untreated control (0 µg/mL w/o stress) set to 100%. Tests were conducted with MTT-Assay (3-(4,5-dimethylthiazol-2-yl)-2,5-diphenyltetrazolium bromide). Bars represent mean values and standard deviations. Data was normally distributed, tested by Shapiro–Wilk test. Statistical significances were evaluated with one-sample *t*-test against control group 0 µg/mL extract. * *p* < 0.05, ** *p* < 0.01, *** *p* < 0.001 (all against 0 µg/mL w/o stress), + *p* < 0.05, ++ *p* < 0.01 (against 0 µg/mL treated with H_2_O_2_ or erastin), *n* = 4 (number of independent experiments).

**Figure 9:**
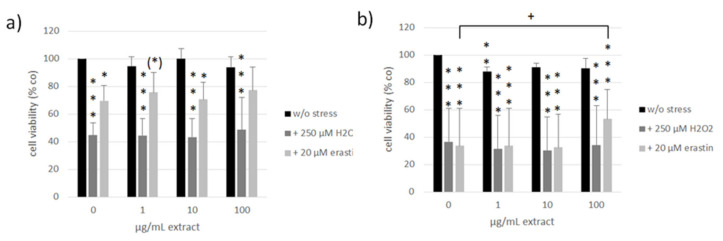
Cell viability of human retinal pigment epithelium (RPE) cell line ARPE-19 treated with different concentrations of ommochromes (1, 10, 100 μg/mL) and/or 250 µM H_2_O_2_ and 20 µM erastin for four (**a**) and 24 h (**b**) in % of the untreated control (0 µg/mL w/o stress) set to 100%. Bars represent mean values and standard deviations. Data was normally distributed, tested by Shapiro–Wilk test. Statistical significances were evaluated with one-sample *t*-test against control group 0 µg/mL extract. (*) *p* < 0.1, * *p* < 0.05, ** *p* < 0.01, *** *p* < 0.001 (all against 0 µg/mL w/o stress), + *p* < 0.05 (against 0 µg/mL treated with erastin), *n* = 4.

ARPE-19 and OMM-1 are established cell models for the assessment of oxidative stress (H_2_O_2_) and ferroptosis (erastin)-induced cell death [64,65,66]. The susceptibility toward these stressors differs between these cell lines [66]. We treated OMM-1 (Figure 8) as well as ARPE-19 (Figure 9) with 1, 10, and 100 µg/mL ommochrome extract to test for possible toxic effects as well as ommochrome extracts and 500 µM H_2_O_2_ (OMM-1) or 250 µM H_2_O_2_ (ARPE-19) to induce oxidative stress or 30 µM erastin (OMM-1) or 20 µM erastin (ARPE-19) to induce lipid peroxidation by ferroptosis. Cell viability was checked after 4 h and 24 h of treatment with MTT (3-(4,5-dimethylthiazol-2-yl)-2,5-diphenyltetrazolium bromide). Data are depicted in % of the untreated control (set to 100%). All data was normally distributed (Shapiro–Wilk test) and statistical significances were calculated with one-sample *t*-test against the control.

In OMM-1, no relevant toxic effects could be detected except for after 1 µg/mL extract treatment after 24 h which reduced cell viability to 85 ± 7% (*p* = 0.034). In ARPE-19, the only significant effect was determined after 24 h of treatment with 1 µg/mL extract which reduced viability to 88 ± 3% (*p* = 0.007). No toxicity was detected at higher concentrations, indicating that these findings are of little biological relevance.

Regarding oxidative stress assays, the viability of OMM-1 was successfully decreased by treatment with H_2_O_2_, which reduced cell viability to 52 ± 12% (*p* = 0.000) and 54 ± 28% (*p* = 0.002) after 4 h and 24 h, respectively. Erastin reduced OMM-1 viability to 76 ± 4% (*p* = 0.002) and 89 ± 7% (*p* = 0.006) after 4 h and 24 h respectively. Ommochrome treatment had no protective effects in this model system but on the contrary even reduced viability in combination with H_2_O_2_ at 100 µg/mL after 24 h with 36 ± 19% (*p* = 0.029). Additionally, 100 µg/mL of ommochrome treatment decreased the viability after four hours of erastin treatment to 65 ± 3% (*p* = 0.009). In ARPE-19 we could find appropriate doses of H_2_O_2_, reducing cell viability to 45 ± 9% (*p* = 0.000) and 37 ± 24% (*p* = 0.000) after 4 h and 24 h as well as for erastin which lowered viability to 70 ± 11% (*p* = 0.018) and 34 ± 27% (*p* = 0.000) at the same time points. We could detect a protective effect of ommochromes after 24 h of erastin treatment at a concentration of 100 µg/mL which increased cell viability from 19% up to 53 ± 21% (*p* = 0.020). Ommochrome extract seem to be promising regarding protection against lipid peroxidation in healthy ocular cells.

### 3.5. Effects on Vascular Endothelial Growth Factor Secretion

**Figure 10:**
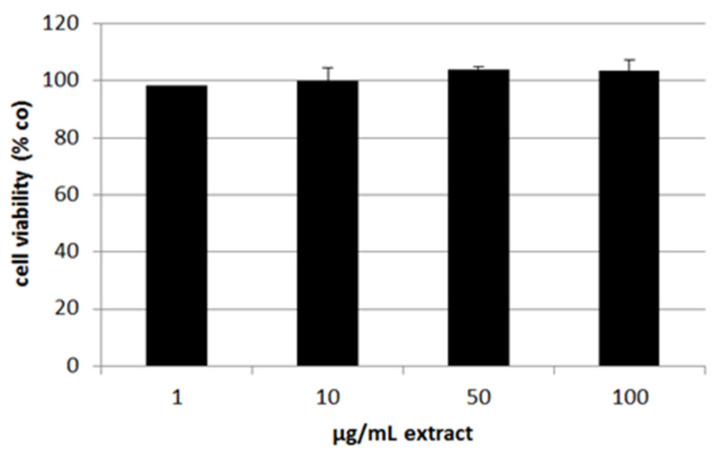
Cell viability of primary porcine RPE cells after stimulating them with 1–100 µg/mL ommochrome extract for 28 days and testing with MTT-Assay. Values are depicted in % of the untreated control set to 100%. No significant effects could be detected (*n* = 3).

Further tests besides oxidative stress protection involved long-term treatment in primary porcine RPE cells. We treated the cells with 1–100 µg/mL extract and measured the cell viability on day 28 with an MTT assay (Figure 10). The extract showed no relevant or significant effects on cell viability after long-term treatment. We also tested time points of three and seven days in RPE as well as three days in ARPE-19 cell line (data not shown). Here, we could also detect no influences on cell viability. We conclude that ommochromes are not toxic or do not influence the cell metabolism. This paves the way for further assays with primary RPE cells.

**Figure 11:**
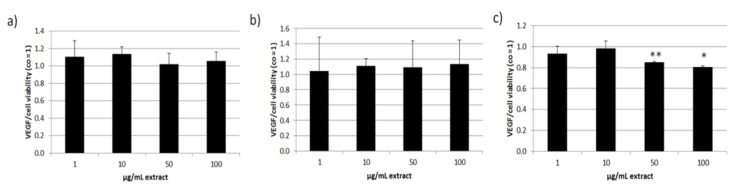
VEGF (vascular endothelial growth factor) secretion of RPE and ARPE-19 cells. Secreted VEGF of supernatants was measured with ELISA (enzyme-linked immunosorbent assay) and calculated as pg/mL and normalized with the corresponding MTT data in % of the control (VEGF/cell viability). This ratio is depicted for ARPE-19 after three days of stimulation (**a**) and for primary porcine RPE after three (**b**) and seven days (**c**) of stimulation, all with 1–100 µg/mL ommochrome extract. Supernatants were collected for 24 h in ARPE-19 and for 4 h in RPE; * *p* < 0.05; ** *p* < 0.01 (versus control), co = untreated control (*n* = 3).

We continued with tests for secreted VEGF in ARPE-19 (three days) and RPE (three and seven days) and normalized the data with the MTT values (Figure 11). In ARPE-19 VEGF secretion was not reduced. Cytokine secretion in RPE was not influenced after stimulating for three days. After one week of stimulation 50 and 100 µg/mL ommochrome extract reduced secreted VEGF to 0.85 ± 0.01 [arb. unit] (*p* = 0.002) and 0.80 ± 0.02 [arb. unit] (*p* = 0.049). Collectively, we could show that ommochromes can reduce this angiogenesis-relevant cytokine depending on the concentration in long-term stimulation.

All data of this chapter was normally distributed (Shapiro–Wilk test) and statistical significances were calculated with one-sample *t*-test against appropriate controls.

### 3.6. Interleukin 6 and Interleukin 8 Secretion

Inflammatory milieu in the eye can be a cause or the consequence for developing an AMD. We tested if the ommochrome extract has anti-inflammatory activities in our model system. We stimulated primary RPE cells with 50 µg/mL ommochromes, as this concentration was found to be effective in cytokine reduction with other marine compounds [65]. For inflammatory stimulation, we treated the cells with three different agents: 1 µg/mL lipopolysaccharide (LPS), 10 µg/mL polyinosinic:polycytidylic acid (Poly I:C) or 50 ng/mL tumor necrosis factor alpha (TNF), respectively, as determined in previous studies [37]. Tests were running for seven days after which we conducted an MTT assay for testing cell viability; also, we measured supernatant after one, three, and seven days to analyze them with ELISA (enzyme-linked immunosorbent assay) for IL-6 and IL-8 secretion as the main indicator for inflammatory activation of RPE cells [37].

**Figure 12:**
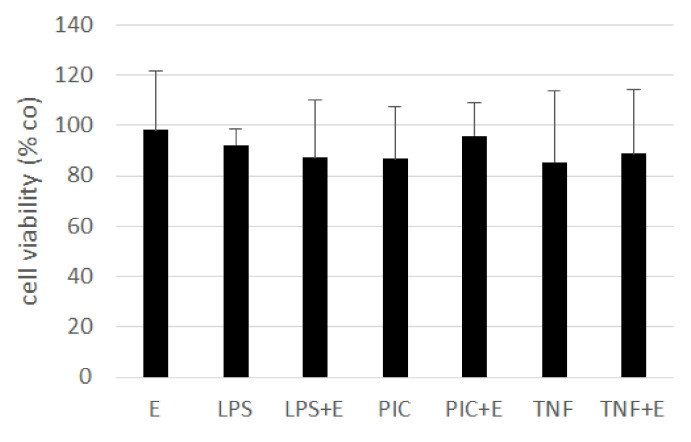
Cell viability of treated RPE cells after seven days. Cells were treated with 50 µg/mL ommochrome extract (E), 1 µg/mL lipopolysaccharide (LPS), 10 µg/mL Poly I:C (PIC), and 50 ng/mL tumor necrosis factor alpha (TNF), and combinations of extract and inflammatory stimulus. An MTT assay was conducted. No significant changes were detected. *n* = 5.

The tests for cell viability showed no significant influences, and no toxic effects could be detected (Figure 12). Therefore, we did not normalize the secreted interleukins with cell viability. The MTT data was normally distributed (Shapiro–Wilk test) and statistical significances were calculated with one-sample *t*-test against appropriate controls.

**Figure 13:**
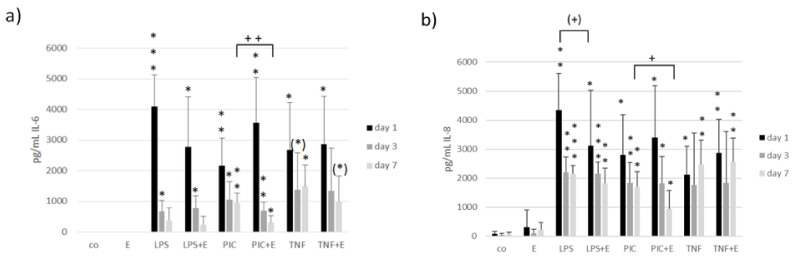
Interleukin 6 (IL-6, (**a**)) and interleukin 8 (IL-8, (**b**)) secretion of RPE cells after one, three and seven days of stimulation with 50 µg/mL ommochrome extract and inflammatory agents, detected with appropriate ELISA. (*) *p* < 0.1; * *p* < 0.05; ** *p* < 0.01; *** *p* < 0.001 (versus control); (+) *p* < 0.1; + *p* < 0.05; ++ *p* < 0.01 (versus inflammatory stimulus without extract).

Concerning tests for inflammatory cytokines we tested again with Shapiro–Wilk test and all data were normally distributed. After that we used an ANOVA (analysis of variance) followed by a consecutive student’s *t*-test to calculate significances. Regarding IL-6 secretion, the LPS (one, three days), Poly I:C (one, three, and seven days) and TNF (one and seven days) controls showed a significant increase in IL-6 secretion (Figure 13). The extract on its own had no effect. Combinations of inflammatory agents and extract showed the following outcome: after seven days we could detect a significant reducing effect after stimulating with Poly I:C and extract with 318 ± 210 pg/mL compared to Poly I:C control with 957 ± 304 pg/mL (*p* = 0.004).

Testing for IL-8 secretion showed similar results. Again, the stress control increased IL-8 secretion successfully (LPS for one, three, seven days; Poly I:C for one, three, seven days; TNF for one and seven days). A tendential decrease in IL-8 after LPS and extract stimulation was detected after one day with 3110 ± 1992 pg/mL compared to LPS control with 4338 ± 1274 pg/mL (*p* = 0.089). After seven days Poly I:C and extract stimulation significantly decreased IL-8 with 942 ± 634 pg/mL compared to 1696 ± 523 pg/mL (*p* = 0.024). Collectively, we could show anti-inflammatory activities of ommochromes depending on the time (day 7) and agent (Poly I:C) which induced the inflammatory response.

## 4. Discussion

Ommochromes are found in the skin of cephalopods where they are involved in the color change of the animal [1]. In addition, they are non-light sensitive pigments present in invertebrate organs of the vision whose function is to filter and absorb light. In conjunction with the optical function, ommochromes play an antioxidant protective role against the blue/violet light [8,9]. Due to their biological activity, these natural pigments can be promising pharmacological preparations for the prevention and treatment of pathologies associated with the development of oxidative stress such as AMD. In this study, we investigated the physicochemical properties and the effect of ommochromes from cephalopod’s skin on the retinal pigment epithelium in regard to oxidative stress, ferroptotic cell death, inflammation, and VEGF secretion.

The color change of ommochrome compounds from octopus extracted with methanol acidified (HCL or acetic) showed strong relation to pH-dependent redox potential. This shows the influence of the number of protons involved in the transfer related to the alteration of the color during solvent extraction. These results coincide with the UV-visible spectra characterized for these compounds [1,2,3,6]. The color variation observed under reductive and oxidative conditions in the different acidified methanol solvents show absorbance maxima (λ_max_) in the UV region in the range characteristic 230–240 nm of the phenoxazone and phenothiazine rings of the ommochrome. In the visible range at 430–520 nm, the differences between pigment are present. A similar yellow ommochrome compound with a UV spectrum showing an absorption peak at 422 nm extracted from the giant squid in ethanol–acetic acid [3,6].

Recent studies have suggested that redox status and anti-radiation capabilities allow phenoxazine compounds to improve oxidative stress. The fact that the ommochrome pigments obtained with MeOH/HCl had twice the antioxidant activity may be due to the reduced nature that favors the extension of their electronic delocalization, with respect to the pigments in MeOH/acetic. Since the antiradical property of these compounds is directly related to the N-H bond, it means that only the reduced ommochromes can act as potent antiradicals and antioxidants. The antioxidant capacity of these compounds was lower than the ascorbic acid used as a control in this study and the antioxidant properties of β-carotene and astaxanthin. Similarly, a moderate activity was reported in the quenching of the ABTS • + radical and a delay in the oxidation of lipids by squid skin extract [2,4].

Thus, ommochromes of squid skin exhibited an inhibitory effect on the BSA fructosylation reaction. It is well known that non-enzymatic glycation (Maillard reaction) under hyperglycemic conditions leads to the formation of so-called advanced glycation end products (AGEs) [63,67]. AGE products, such as advanced lipoxidation end products (ALEs), are toxic to cells. The formation and accumulation of AGE products in various cells and tissues leads to damage to extracellular and intracellular structures, disruption of their functions and is one of the causes for the development of diabetic complications. Increased accumulation of AGE products has been revealed in aging, diabetes [68], arthritis, atherosclerosis, chronic renal failure, nephropathy, neuropathy, Alzheimer’s disease, and other pathologies [69]. The ability of squid skin ommochromes to suppress the process of nonenzymatic glycation may be associated with their antioxidant activity [8,10]. It is well known that antioxidants can inhibit the development of the Maillard reaction [63]. The discovered property of squid ommochromes to inhibit the glycation process can be used in pharmacological practice.

In our study, we could not detect a general anti-oxidative effect of the tested ommochrome in a cellular context, as it neither protected the RPE cell line, ARPE-19, nor the uveal melanoma cell line OMM-1 from H_2_O_2_. However, we could find a significant protective effect against erastin, which is a compound-inducing ferroptosis as found in lipid peroxidation [18]. This is in accordance with the proposed effect of ommochromes against lipid peroxidation [9]. It has to be pointed out, however, that the effect of the tested ommochrome was time and concentration dependent and was only found at the highest concentration tested. As ommochromes are presumed to be strong antioxidants, this finding may be considered surprising. However, retinal pigment epithelial cells, especially when used in confluence, are highly resistant against oxidative stress and the high intrinsic activity in their protective oxidative stress pathways may hinder externally induced oxidative stress protection [31,70,71]. Indeed, similar results have been obtained with other antioxidant compounds such as fucoidan that only displayed modest effects on oxidative stress-induced cell death in ARPE-19 cells [65,66,72]. Therefore, the effect of ommochromes against erastin-induced cell death does indicate a protective potency of this compound.

In addition to oxidative stress, inflammation is a major contributor to AMD pathology [21,30]. To the best of our knowledge, the effect of ommochromes on inflammation has not been investigated so far. We induced the secretion of pro-inflammatory cytokines by treating the RPE cells with LPS, Poly I:C, or TNFα. We have shown previously that these stimuli in the utilized concentrations induce IL-6 and IL-8 secretion in RPE cells [37]. The three stimuli exert their effect via different pathways, with LPS being an agonist of TLR-4, Poly I:C of TLR-3 and TNFα being a proinflammatory cytokine presumably acting via TNF Receptor 1 [73,74,75]. Interestingly, ommochromes reduced the secretion of both IL-6 and IL-8 in RPE cells stimulated with Poly I:C, but not in cells stimulated with LPS or TNFα, indicating a specific effect in TLR-3-mediated pro-inflammatory signaling. Further research is warranted to further elucidate the exact mechanisms of this reduction. TLR-3 activation has been implicated in the pathogenesis of AMD [38], and it will be of great interest to investigate the influence of ommochromes on other TLR-3 induced effects in the RPE, such as TLR-3 induced VEGF secretion, TLR-3 induced cell death, or TLR-3 induced loss of barrier function [39,49].

VEGF secretion is the major pathogenetic factor for the development of exudative AMD [29]. RPE cells constitutively secrete high amounts of VEGF [65,76]. To the best of our knowledge, the effect of ommochromes on VEGF secretion has not previously been investigated. Our study revealed that a long-term stimulation (7 days) with ommochromes resulted in a significant reduction in VEGF. As this effect is not seen after a shorter time period, the effect cannot be explained by steric inhibition caused by a binding of ommochromes at VEGF (as found in traditional VEGF antagonists [77]). Instead, these data indicate an effect of the ommochromes on protein expression that results from long-term exposure. Further research needs to be conducted to decipher the interaction of ommochromes with these cells.

In order to reduce the variability of bioactivity due to source and extraction methods, synthesized ommochromes might be of great interest for further development [78]. However, in order to synthesize the most appropriate ommochrome for this use, further research is warranted and a thorough investigation of the bioactivity and interaction of the ommochromes with the RPE cells needs to be conducted. In addition, the bioavailability needs to be determined. Here, the low solubility of the ommochromes may be a challenge.

In summary, our study shows a protection of RPE cells against erastin-induced cell death in RPE cells and, to the best of our knowledge, for the first time, a reductive effect of ommochromes from cephalopod skin on pro-inflammatory and pro-angiogenic cytokines in the RPE. Given that oxidative stress, inflammation, and angiogenesis are very important pathogenic factors in AMD, these findings suggest ommochromes as an interesting substance for further research and possible new therapy development. More research is needed to understand the nature of the interaction between ommochromes and RPE cells.

## 5. Conclusions

The aim of this study was to investigate ommochromes from skin of cephalopods regarding promising effects in ocular cells. We examined biological activities that could be relevant for AMD such as cell viability, oxidative stress protection, influence on VEGF secretion, and inflammation. Cell viability was not relevantly influenced in any cell model. We showed protective effects in ARPE-19 against erastin-induced ferroptosis. In contrast, cell death increasing effects after oxidative stress induction were detected in OMM-1. We showed VEGF-inhibiting effects in primary porcine RPE cells and anti-inflammatory effects by the reduction in secreted interleukin 6 and interleukin 8. The effects are concentration and time-dependent. We could show promising effects of cephalopods ommochromes in ocular cells rendering them as valuable substances for further research in the field of ophthalmology, especially for AMD.

## Data Availability

Not applicable.
